# Protein-Protein Interface Detection Using the Energy Centrality Relationship (ECR) Characteristic of Proteins

**DOI:** 10.1371/journal.pone.0097115

**Published:** 2014-05-15

**Authors:** Sanjana Sudarshan, Sasi B. Kodathala, Amruta C. Mahadik, Isha Mehta, Brian W. Beck

**Affiliations:** 1 Department of Biology, Texas Woman's University, Denton, Texas, United States of America; 2 Department of Mathematics and Computer Science, Texas Woman's University, Denton, Texas, United States of America; 3 Department of Chemistry and Biochemistry, Texas Woman's University, Denton, Texas, United States of America; University of Copenhagen, Denmark

## Abstract

Specific protein interactions are responsible for most biological functions. Distinguishing Functionally Linked Interfaces of Proteins (FLIPs), from Functionally uncorrelated Contacts (FunCs), is therefore important to characterizing these interactions. To achieve this goal, we have created a database of protein structures called FLIPdb, containing proteins belonging to various functional sub-categories. Here, we use geometric features coupled with Kortemme and Baker's computational alanine scanning method to calculate the energetic sensitivity of each amino acid at the interface to substitution, identify hotspots, and identify other factors that may contribute towards an interface being FLIP or FunC. Using Principal Component Analysis and K-means clustering on a training set of 160 interfaces, we could distinguish FLIPs from FunCs with an accuracy of 76%. When these methods were applied to two test sets of 18 and 170 interfaces, we achieved similar accuracies of 78% and 80%. We have identified that FLIP interfaces have a stronger central organizing tendency than FunCs, due, we suggest, to greater specificity. We also observe that certain functional sub-categories, such as enzymes, antibody-heavy-light, antibody-antigen, and enzyme-inhibitors form distinct sub-clusters. The antibody-antigen and enzyme-inhibitors interfaces have patterns of physical characteristics similar to those of FunCs, which is in agreement with the fact that the selection pressures of these interfaces is differently evolutionarily driven. As such, our ECR model also successfully describes the impact of evolution and natural selection on protein-protein interfaces. Finally, we indicate how our ECR method may be of use in reducing the false positive rate of docking calculations.

## Introduction

Proteins interact with and bind to other proteins forming both transient and long-term networks of specific complexes whose interfaces have highly-specific amino acid interactions [1]–[6]. These interfaces play vital roles in biological functions such as signal transduction, enzyme and immune regulation, adhesion, force generation, and maintenance of cellular structure. Methods for the identification and characterization of protein-protein interactions (PPIs) are thus critical to understanding how living systems function.

Development of experimental and computational techniques to identify PPIs has shed light on the determinants of specific interactions, as well as on some general features for different types of interactions [2]–[5], [7]–[13]. Experimental high throughput screening methods [3]–[5], [16] have provided information to construct large databases [17]–[19] of PPIs and related functions. Computational methods such as molecular modeling and docking, have generally identified the shape, electrostatic complementarity, buried surface area, flexibility, solvation energy, and sequence conservation of the interactors (amino acid residues) as key features in interface detection [6], [7], [11]–[13], [20]–[23]. Use of these known relationships to better elucidate the principles by which amino acids are positionally organized and thus contribute energetically to interfaces would allow specific structure/function relationships to be characterized. Such knowledge could also promote the finding of novel interfaces via computational docking calculations, as well as allowing the testing of rival protein structure/function hypotheses. Unfortunately, the different attempts at characterization continue to be hampered by a fundamental lack of understanding about the underlying geometric and energetic principles of amino acid interaction across protein interfaces [6], [8], [15], [21], [24]–[26].

Several potential reasons for this exist. Both experimentally and computationally, it has been observed that few of the residues present in a PPI are essential for maintenance of the integrity of the interface [2], [8], [15]. Some success has been had identifying these important “hotspots”, particularly with computational alanine scanning methods (CAS) [2], [27]–[31]. However, the use of CAS in PPI detection has had mixed success. CAS methods often very accurately distinguish residues critical to known interfaces, while failing to identify all the residues in an interface [15]. Ofran and colleagues suggest that this may be due, in part, to a bias towards hotspot residues that may treat non-hotspot residues as “noise” and thus fail to identify all the residues in a PPI [15].

An additional reason PPI principles may be difficult to elucidate can be found in how the experimental data used to develop computational methods like docking is organized and utilized. Most data for the patterns of amino acid characteristics at PPIs come from atomic resolution structures of protein complexes deposited at the Protein Data Bank (PDB) [32]. While an understanding of PPI principles for both prediction and design necessitates the use of natural exemplars, whether a reference structure is a highly specific interaction used in nature and critical for a biological function or whether the association is the result of the experimental conditions used in the technique can often be unclear. The majority (approximately 80%) of PPI structures available from the PDB are obtained through X-ray crystallography [33]. The very symmetrical and tightly packed structures that promote facile structure determination can also indicate interfaces not present in the cellular milieu [26], [34], [35]. As with hotspot/non-hotspot bias, development of PPI predictive methods based simultaneously on both aggregative (e.g. crystal contacts) and functionally-linked PPIs may obscure trends such that both can fail to be identified.

Several groups have classified PPIs into different operationally defined categories such as, homo- and hetero- complexes, obligate and non-obligate complexes, and transient and permanent complexes (reviewed in [6], [36]). These categories, however, often mix structural and functional properties in their operational definitions. While structure and function are, of course, related, natural selection operates on biological function, and it may serve useful to identify the functional importance of a given PPI as a separate characteristic feature. In this work, we operationally define Functionally-Linked Interfaces of Proteins (FLIP), and the residues forming these interfaces, to be those for which mutation or other chemical modification has been found to alter the native biological function. Similarly, we define PPIs that do not have such a known alteration in function as Functionally uncorrelated Contacts (FunC).

Separation of FLIPs from FunCs can be problematic using PDB data alone, and additional knowledge is generally required [7], [13], [37] FLIPs and FunCs can be thought of as positive-design (specific) and negative-design (aggregative) natural exemplars in the parlance of Havranek [38]. While the PDB often provides a “Biological Assembly” structure (BioUnit) in addition to the standard “Asymmetric Unit” structure, in our experience, the correlation of the BioUnit structures with FLIPs is not straightforward. BioUnits are often not available, are duplicates of the Asymmetric Unit with little justification for that assignment, or are specified for non-native interactions as in the case of rabbit actin with bovine DNase (PDBid: 1ATN). As mentioned previously, shape and electrostatic complementarity, buried surface area, flexibility, solvation energy, amino acid composition, hydrophobicity, and sequence conservation are all common used features use to characterize and predict the quaternary assemblies and improve estimation of likely solution state structures [7], [11]–[13], [22], [39]. Indeed, more recent BioUnit assignments have been improved through the automated use of tools like PISA, which has a particular strength in that it leverages solvation energy calculations in addition to other features to identify macromolecular complexes in solution [13]. Even with these enhanced analyses, the relationship of the complex with function may still be problematic. For example, PISA, NOXclass, and EPPIC servers all identify Actin:DNase as the likely BioUnit [11]–[13]. As a result, the ability to distinguish FLIP from FunC, though improved, remains obscure. While large interactome databases exist that often do indicate functional correlation [17]–[19], they generally specify whole protein chain or complex interactions and do not specify data at the atomic level.

In principle, it is possible to use atomistic or coarse-grain computational methods, including docking methods, that use generic, empirical amino acid interaction functions to successfully predict quaternary interactions [21], [31], [40]–[42]. Unfortunately, two problems generally arise: 1) the false positive rate (average number of predictions needed to obtain a structure similar to a natural exemplar) is fairly high [14], [40], [43] and 2) while accurate structures can be identified, assessment as to the functional significance (i.e. FLIP or FunC) is not generally identified or remains obscure [14], [21], [26], [40].

Physico-chemical properties of the amino acid residues in PPIs other than sensitivity to alanine substitution have also been investigated, including hydrophobicity, amino acid composition, hydrogen bonding potential, sequence conservation, and solvent accessible surface area (SASA), all with differing success [6], [14], [26]. Combining these methods in hybrid approaches has improved successful identification of native PPIs relative to any one property alone [6], [11], [13], [26].

**Figure 1:**
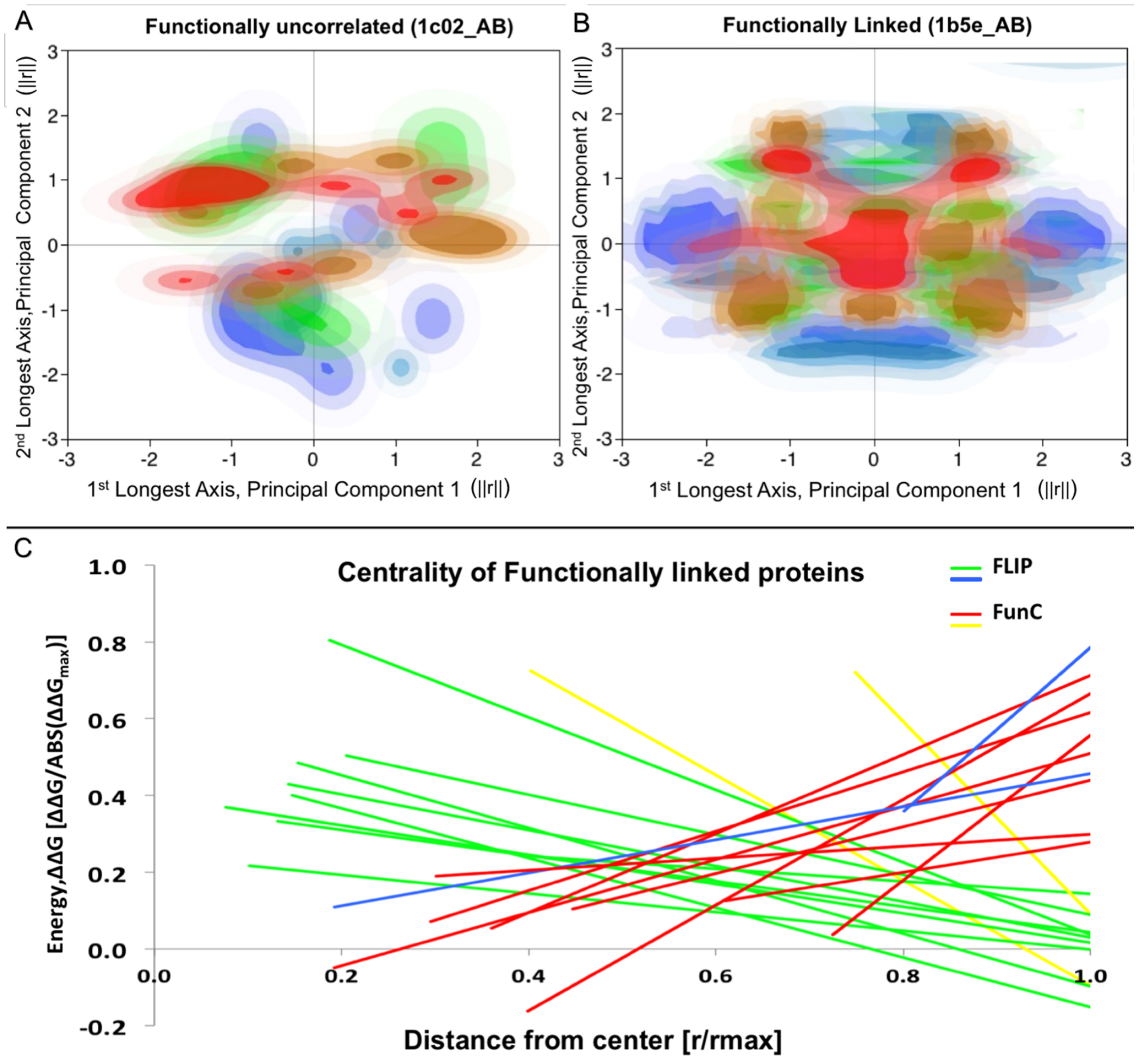
Distribution of alanine substitution energies in FLIP and FunC interfaces. (a) and (b) show a histogrammed contour plot colored blue-to-red of the ΔΔGala of substitution to alanine of interfacial residues (blue: more favorable values, red: more disruptive values). The plot axes are the first two principal components of the geometric distribution of alanine Cα positions. PCA was used to align the interface along the X- and Y-axes. Axes are normalized. (a) ΔΔGala of the FunC interface from PDBid: 1c02, chains A&B. (b) ΔΔGala of the FLIP interface from PDBid: 1b5e_AB, chains A&B. (c) Linear regressions of ΔΔGala vs. Distance from interface center. Regressions for the interfaces in the FLIPdb training set with the 10 most positive [1acy_HP, 1biq_AB, 2cii_AC, 1b5e_AB, 1edh_AB, 1pky_BD, 1tx4_AB, 1hjc_AC, x1bsf8_AJ, 1bo5_OZ] and 10 most negative [1tzi_AV, 1acy_LP, x1ppf2_EZ, x1dv82_AC, x1wtl_BZ, x1py94_AE, x1erv2_AC, x1gaf2_LY, 1scu_BD, 1c02_AB] intercepts. FLIP are shown in green and blue [1tzi_AV, 1acy_LP]. FunC are shown in red and yellow [x1bsf8_AJ, 1bo5_OZ]. ΔΔGala are normalized to MAX(ABS(ΔΔGala)), while distances of each residue's Cα from the mean of the Cα positions (Center of Interface) are normalized to MAX(distance). All 3 plots generally show that FLIP interfaces are more centralized and radially symmetric than FunC interfaces. 80% of shown positive intercepts are FLIP and 80% of shown negative intercepts are FunC. [*Figures (a,b) generated using JMP* [*46*] *. Figure (c) generated using Microsoft Excel, 2008*]

**Figure 2:**
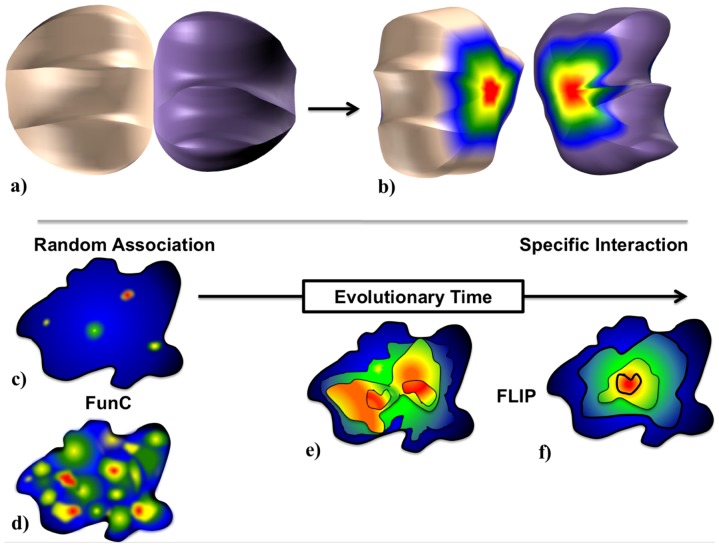
The Energy Centrality Relationship (ECR) for interface evolution. The ECR hypothesis is that upon initial fortuitous protein-protein association, residues in a nascent interface have a selective pressure to maintain or improve the affinity arising from the initial contact, while simultaneously having a similar pressure on residues surrounding that contact. (a) and (b) show a conceptual PPI that has a radially symmetric distribution of ‘hot’ (energetically favorable, red) and ‘cold’ (energetically unfavorable, blue) residues in a FLIP, while (c) and (d) are example residue energy distributions of weaker (c) and stronger (d) affinity FunC. Over evolutionary time (c–f), selective activity, affinity, and specificity pressures on residues in a FunC produce a radially symmetric pattern in the energetics of the interface. The resulting interface should demonstrate “stronger” energies near the “older” regions of the interface. These “older” regions may or may not demonstrate sequence conservation as the pressure is on energy, not identity. As natural interfaces are generally more punctate than the ideal model, we expect that while both FLIP and FunC interfaces may demonstrate multiple contacts, only FLIP interfaces will maintain overall centrality (e–f).

**Figure 3:**
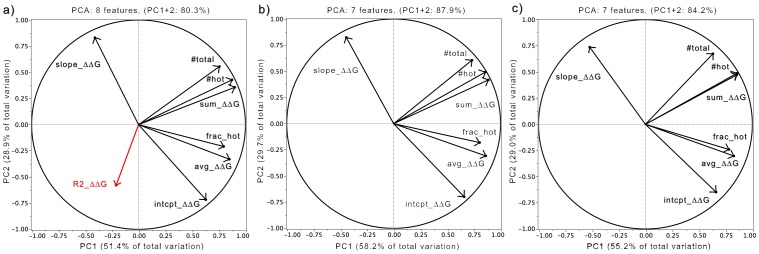
Correlation of Features with Principal Components. Loading plots of the eigenvector coefficients of each feature analyzed by PCA show the influence and correlations of each variable to the principal components. Eight features were analyzed to identify the set of features that could represent ∼80% of data variation in the first two principal components (see text for feature descriptions). (a) 80.3% of the total variance of all eight features could be accounted for with just the first two PCs, though R2_ΔΔG (red) had demonstrably smaller coefficients. (b) Exclusion of R2_ΔΔG produced a PCA over 7 features whose PC1 and PC2 accounted for 87.9% of the variance. (c) After removal of 49 interfaces predicted to be FLIP in the first PCA, a second round of PCA using the same seven features but with only data for the remaining 110 protein interfaces was calculated. This PCA produced eigenvectors that had 84.2% of the variance in the first two PCs. [*Figure generated using JMP* [*46*] *and Microsoft Excel, 2008*].

**Figure 4:**
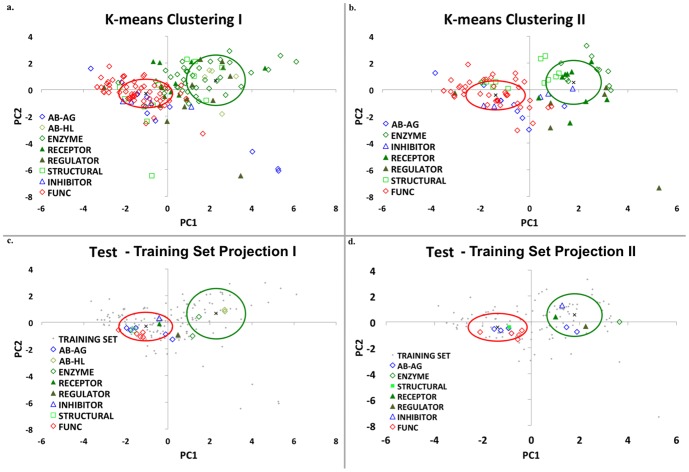
PCA and K-means clustering of Training and Test-18 sets. Principal component analysis followed by K-means clustering was performed on the residues in the 100 FLIP and 60 FunC interfaces in the FLIPdb. The same 7 features identified in Figure 3 are used here and the number of clusters was set to k = 2. Green (“cluster 1”) and red (“cluster 2”) ovals represent 1 standard deviation for Euclidean distances around the cluster centroid marked by “**x**”. Interfaces are indicated with symbols representing their functional sub-category. Green and Blue symbols are FLIP structures, but blue symbols are specifically AbAg and Inhibitor sub-categories. Red symbols are FunCs. (a) and (b): training set. (c) and (d): Test-18 testing set. (a) 49 FLIP interfaces (mostly enzymes and immunoglobin Heavy-Light chains) and 1 FunC are identified in cluster 1 (98% *precision*). (b) After removal of these 50 interfaces, a second PCA analysis of the remaining 110 interfaces produces new clusters with 48 and 62 members, respectively. PCA 2 Cluster 1 is 64% FLIP and cluster 2 is 68% FunC. Overall accuracy across both (a)+(b) is 76%. (c) and (d) show the projection of the 7 feature values 18 unrelated PPIs in the Test-18 set through the principal components developed on the training set. Enzymes and immunoglobin Heavy-Light again dominate cluster 1 (100%) and overall accuracy in both clusterings is 78%. [*Figure generated with JMP* [*46*] *and Microsoft Excel, 2008*].

In light of these improvements, a hybrid approach that includes the statistical analysis of (a) atomic-resolution interface geometries and (b) CAS-based energy data of protein structures pre-classified based on functional importance (FLIP/FunC) may be successful, both in improving detection of interfaces and increasing our understanding of general principles of interface formation. To test this concept, we collected a set of PPI structures available in the PDB starting from a subset of members of commonly used sets to test PPI and docking software [7], [22], [44]–[46], and added additional structures of interest to the lab. We then used additional literature sources to manually categorize the interfaces as being FLIP or FunC (FLIPdb, see Methods). For each interface in FLIPdb, we used Baker's CAS method [30] and our own geometry calculations (see Methods) to determine the energetics of alanine substitution of residues in a PPI as a function of geometric distribution in the interface. No attempt was made to bias towards only hotspot data. Using Principal Component Analysis [47] and K-means clustering [48] we were able to identify seven physical characteristics that could distinguish FLIP interfaces from FunC interfaces with 76% accuracy. These same characteristics, when tested against a set of 18 unrelated PPI structures and a subset of 170 PPI from the set of Dey et al., were also able to distinguish FLIP from FunC with 78–80% accuracies. Overall, FLIP interfaces appear to have greater overall sensitivity to ala substitution than FunC (Figures 1–4), *particularly toward the center of the interfaces*. This may be related to the finding that cores of interfaces have greater sequence conservation, than interfaces rims [49]. Both are consistent with the ideas that FLIP interfaces are more specific than FunC interfaces [1], [6] and that they may evolve increasing specificity radially across a PPI over evolutionary time (Figure 2a–c).

The novelty of this approach, which we term the Energy Centrality Relationship (ECR), is that through the combination of geometric and energetic data, we are able to not only reproduce functional classifications, and describe physical chemical sources of these differences, but also have a model that is consistent with natural selection pressures on protein interfaces.

## Results

### Database Composition, FLIPdb

After construction, our FLIPdb database referenced 160 PPIs between 233 protein chains that were contained in 94 PDB structural files. This set was categorized and divided into 100 FLIP interfaces and 60 FunC interfaces. We further sub-categorized these PPIs into 7 FLIP and 2 FunC sub-categories: 1) antibody-antigen (AbAg); 2) immunoglobin Heavy Chain/Light Chain (AbHL); 3) Enzyme-Enzyme, both transient and persistent (Enzyme); 4) having a generally persistent structure that provides mechanical stability, such as cytoskeletal or viral proteins (Structural); 5) peptide/protein inhibitors to an enzyme (Inhibitor); 6) proteins whose function is to recognize peptides/proteins (Receptor); 7) proteins regulated by post-translational modification by another protein (Regulated); 8) PPIs in an asymmetric crystal unit NOT found to be FLIP (FunC); and 9) PPIs obtained by applying crystal symmetry transforms to FLIP structures (XFunC). This set of PPIs (see supplementary Table S1) was used for training and development (summary in Table 1).

**Table 1:** Summary of protein and protein interface counts in FLIPdb.

Function		Training Set			Test 18 Set		
Category	Sub-categories	PDB Structures	Protein chains	Interfaces	PDB Structures	Protein chains	Interfaces
FLIP	AbAg*	4	15	12	1	6	5
	AbHL*	5	10	5	1	4	2
	Enzyme	33	74	40	2	4	2
	Structural	7	21	16	1	2	1
	Receptor	7	16	10	1	2	1
	Regulator	9	20	12	1	2	1
	Inhibitor	3	10	5	1	2	1
	Total	63	155	100	7	18	13
FunC	FunC	22	47	25	-	-	-
	XFunC‡	23	44	35	5	10	5
	Total	44	89	60	5	10	5
Total		94	219	160	7	19	18

* Proteins chains are common to multiple sub-categories though the interfaces are distinct.

‡ Interfaces are constructed from existing FLIPs through coordinate transformations arising from the symmetry of the source X-ray crystal structure (XFunCs).

FLIPdb contains 160 interfaces in 94 structures involving 219 individual protein chains. These interfaces have been assigned to FLIP or FunC functional categories and 9 functional sub-categories based on a review of the literature (see Supplement Table S1). Due to the reuse of some chains, the totals represented in the first two columns do not sum across sub-categories.

An additional set of 18 PPIs between 19 protein chains in 7 PDB files was also categorized into 13 FLIP and 5 FunC interfaces and sub-categorized as above (see supplementary Table S1). This second set of PPIs was comprised of proteins that were generally less than 70% sequence identical to proteins in the training set and was used for cross-validation testing (Table 2).

**Table 2:** Accuracy of clustering in Training and Test-18 sets.

**Training Set** †	**True Positive (TP)**	**False Positive (FP)**	**False Negative (FN)**	**True Negative (TN)**	**Accuracy**	**MCC**
	**1^st^ clustering**					
	49	1	51	59	67.5%	0.49
	**2^nd^ Clustering**					
	31	17	20	42	66.4%	0.32
**Total**	80	18	20	42	76.3%	0.50
**Test 18 Set** †	**1^st^ clustering**					
	3	0	10	5	44.4%	0.28
	**2^nd^ clustering**					
	6	0	4	5	73.3%	0.58
**Total**	9	0	4	5	77.8%	0.62

†) TP: FLIP found in Cluster 1TN: FUNC found in Cluster 2

FP: FUNC found in Cluster 1FN: FLIP found in Cluster 2

The accuracy and Matthews correlation coefficient (MCC, a measure of the quality of a binary classification) of the results of the clusterings shown in Figure 4 are indicated. The overall accuracy is 76% and 78% for both training Test-18 sets, respectively. TPs are quite readily identified in both training and Test-18 sets (80% and 69% *sensitivity*, respectively). The majority of TPs are enzymes and immunoglobin heavy chain-light chain interactions. TNs are less well identified (70% and 56% *negative predictive values*, respectively). MCCs of 0.50 and 0.62 indicate that our simple two-category approach is generally appropriate.

Finally, a third set of 170 PPIs between 301 protein chains in 139 PDB files was examined. These 170 PPIs represent a subset of 54% of the weakly and strongly interacting PPIs characterized by Dey and colleagues [7]. This set was not rigorously curated as to FLIP/FUNC status so as to compare the results of our training set with that of Dey. Overall, the structures and energetics of 348 PPIs were categorized and examined.

### CAS ΔΔG distribution in PPI

We used Baker's CAS method [30] coupled with our own software to determine the sensitivity to alanine substitution of residues in a PPI, as a function of geometric distribution in the interface. All geometric analyses were based on residue Cα positions. This sensitivity was compared between FLIP and FunC PPIs in the FLIPdb. Two representatives of this are shown in Figure 1, in which we compare a FunC (Yeast Phosphotransferase Ypd1p, PDBid: 1C02) and a FLIP (T4 bacteriophage dC-hydroxymethylase dimer, PDBid: 1b5e). Histogrammed contours of the pseudo-free energy change upon alanine substitution (ΔΔG) are plotted on the principal component analysis (PCA) projections of the interface residue geometry (Figures 1a,b). (Note, that in this work, we follow Baker's use of the terms “free energy” and “ΔG” for consistency with the software output.) These distributions indicate that in the FLIP, “hotter” residues (whose CAS analysis resulted in more PPI destabilization upon substitution) tend to be more centrally located and tend to show a progressive radial symmetry. In contrast, the “hotter” residues in the FunC are fairly evenly distributed throughout the interface. Some “cold” residues (those favoring Ala substitution) are found near the interface center. These CAS energy distributions are representative of other FLIPs and FunCs. When all the ΔΔG vs. distance from the Center of Interface (CoI) were then fitted to a 1st order polynomial line via linear regression, 8 of the 10 highest intercepts were found to be FLIP, while 8 of 10 lowest intercepts were found to be FunC (Figure 1c). In general, FLIPs were found to fit a line better than the FunC (coefficients of determination, R^2^, were an order of magnitude larger). The FLIPs were also found to generally have a negative slope, indicative of a central tendency, whereas the FunCs generally had near flat or small magnitude positive slopes. The small magnitude slope and poor R^2^ suggests little geometric central tendencies in the FunC. These trends were generally maintained throughout FLIPdb, with most FLIPs having a radially symmetrical central tendency and most FunCs demonstrating little-to-no correlation with distance from the center of the interface. One-way pairwise ANOVA at an **α** = 0.10 analyzing the slopes and intercepts indicated that the differences between FLIP and FunC were significant with P<0.0006 and P<0.09, respectively.

### Energy Centrality Hypothesis

There is no *a priori* reason FLIP PPIs should demonstrate a central tendency relative to FunC PPIs. Unless an organizing principle was involved, one might expect an interface to have a random correlation between CAS ΔΔG and geometry (Figure 2c–d). The presence of such a central tendency (Figure 1) in FLIP interfaces suggests that they are indeed organized (Figure 2e–f), perhaps through a natural selection process (see Discussion and Figure 2).

### Energetic and Geometric Features

Though PPIs are complex 3-dimensional entities, for the sake of simplicity of analysis, we unified CAS ΔΔG and structural geometry characteristics into scalar quantities that could be used to describe a PPI. Three features arose from the regression of energy to geometry: the rate of change of substitution energy as a function of distance (Δr) from the interface center (slope_ΔΔG), the extrapolated maximum ΔΔG sensitivity at the interface center (intcpt_ΔΔG), and the adherence of the ΔΔG and Δr data to a linear relationship (coefficient of determination, R2_ΔΔG). Three features were found that describe the net sensitivity of an interface to CAS: net sum of all ΔΔG changes (Sum_ΔΔG), mean ΔΔG for all interface residues (Avg_ΔΔG), and total number of residues in the interface (#total). The remaining two features address the number of residues extremely sensitive to Ala substitution (“hot” residues, residues with ΔΔG larger than +1 kcal/mol): the number of hot residues (#hot), and the ratio of hot to total (frac_hot). One-way pairwise ANOVA at an **α** = 0.10 indicated that all features except R2_ΔΔG were significantly different between FLIP and FunC with #hot, total, Sum_ΔΔG, frac_hot, and Avg_ΔΔG having P<0.0001, intcpt_ΔΔG having P<0.0006, and slope_ΔΔG having P<0.09. Since these features could reasonably be viewed as coupled, we also performed one-way ANOVA with repeated measure at an **α**  = 0.10 and with Tukey-Kramer post-hoc analysis. This analysis indicated differences between FLIP and FunC for #hot, total, Sum_ΔΔG that were significantly different with P<0.0001. Though shown to be statistically different, individually none of these features were found to sufficiently correlate with FLIP or FunC categories such that a single feature could be used to identify the category.

### Principal Component Analysis and K-Means Clustering

When no single feature could easily discriminate FLIP from FunC, yet each feature yielded significant differences between groups, the multi-factoral approach of PCA was used. Initial PCA analysis of the 8 features for all 160 PPI in the training set yielded a set of principal components (PCs) that reproduced 80% of the normalized data variation in the first two PCs (Figure 3a). Analysis of the eigenvector coefficients (Figure 3a) agreed with the ANOVAs indicating that the variance in the data was far less dependent on a strict adherence to a 1^st^ order linear model. Thus, for all subsequent analyses, R2_ΔΔG was dropped as a feature. The resultant 7-feature PCA reproduced 88% of the remaining data variation in the first two PCs (Figure 3b). Subsequent K-means cluster analysis with a two-cluster assumption of this data (Figure 4a), produced two clusters whose centroids straddled the origin for both PC1 and PC2 indicating opposing correlation trends. Analysis of these clusters revealed they had high *precision* and *specificity*. Cluster 1contained 49% of all FLIPs but only 2% of FunC PPI (Table 2). Cluster 2 contained 51% of all FLIPs and 98% of FunC PPI. The FLIP PPI in cluster 1 were predominately in the Enzyme (72% of Enzyme) and Antibody-Heavy/Light sub-categories (100% of AbHL). Cluster 2 was dominated by FunC/XFunC (98% of FUNC), Antibody-Antigen (75% of AbAg), and Inhibitor sub-categories (100% of Inhibitor). Closer examination of cluster 2 revealed that FLIPs assigned to this cluster tended towards more positive PC1 values and larger magnitude PC2 values than FunCs/XFunCs. This consistency in trend suggested a second PCA over the same features might provide further distinction between FLIPs and FUNCs. A new PCA of only the 110 PPI in cluster 2 of the first PCA produced new PCs with extremely similar eigenvector coefficient correlations to the first PCA (Figure 3c). The same set of features still produced PCs that represented 84% of the resultant data variation in the first two PCs. This confirmed that similar data dependencies were in effect between the two PCA. K-means clustering of this second PCA again produced 2 clusters that straddled the origin for both PC1 and PC2 (Figure 4b). As with the first PCA, cluster 1 of the second PCA was predominately FLIP, containing 61% of the remaining FLIPs but only 28% of the total FunCs. Likewise, cluster 2 was predominately FunCs, containing 20% of the FLIPs and 70% of the FunCs PPI (Table 2). Over two-rounds of PCA, 80% of the FLIPs were found in the clusters positively correlated with the features, and 70% of the FunCs were found in clusters negatively correlated with the features.

### Accuracy and Matthews Correlation

Analysis of the two rounds of PCA of the training set PPI data indicated that the overall accuracy (the propensity to correctly identify FLIP or FunC) was ∼67% in each PCA round. Over both rounds of PCA, the accuracy was 76% (Table 2). The Matthews Correlation Coefficient, a measure of how well a binary classification matches the data, was 0.49 in PCA round one, 0.32 in PCA round two, and 0.50 across both rounds. Such MCCs indicate a two-category assumption is quite consistent with the data.

### Cross-validation Testing

While analysis of the training set data very favorably predicted distinct feature set correlations between FLIPs and FunCs, it was possible that the relationship was training set dependent and demonstrated compositional bias. In order to test this, we undertook three types of cross-validation testing: validation on two test sets and random sub-sampling validation on the training set.

We first repeated the analyses on the 18 member test set (hereafter, Test-18). The additional interfaces in this set were between protein chains that generally had less than 70% sequence identity to chains in the training set (Table S1, see Methods). No new PCA or K-means clustering was undertaken; rather the features of Test-18 were projected through the PCs of the training set. Test-18 projections are shown in Figures 4c,d. As with the training set, FLIPs in the Enzyme and Antibody-Heavy/Light sub-categories could be reliably identified in cluster 1 of PCA round 1. Similarly, FunCs dominated the composition of cluster 2 in PCA round 2. While the accuracies of the PCA 1 projection were disappointingly lower than the training set (48%), the 2^nd^ round projection accuracies were larger (73%), and the overall two-round accuracy was actually slightly higher than the training set at 78% (Table 2). Similarly, MCC values were also slightly higher, at 0.62 (Table 2). This backhanded success may in part arise due to the relatively high fraction of AbAg in Test18, as AbAg are generally identified in round 2.

We next repeated the analyses on a second test set of 170 PPI derived from the dataset of Dey and colleagues (see Methods) [7]. The dataset of Dey and colleagues was designed to analyze PPI known to interact weakly or strongly in solution. Our subset (hereafter Dey-170) represents about 54% of the full Dey dataset and contains 32 weakly interacting PPI (weak) and 138 strongly interacting PPI (strong) (Table S2). Dey-170 was not rigorously curated as to FLIP/FUNC status but instead was used to examine two model assumptions: a) Assume all 170 PPI are FLIP-like since all are known to oligomerize in solution or b) Assume weak PPI are more FUNC-like and strong PPI are more FLIP-like. Testing these assumptions allows us to examine how well our operationally defined categories of FLIP and FUNC agree with the weak and strong PPI characterized by Dey. Again, the values of the 7 features of each Dey-170 PPI were projected through the PCs of the training set (Figure S1a,b, Table S3). In projection round 1, cluster 1 contained 59% of the strong PPI and no weak PPI. Cluster 2 contained 100% of the weak PPI and 41% of the strong PPI. In round 2, 75% of the remaining strong PPI and 38% of the weak PPI were found in cluster 1, while 62% of weak PPI and 10% of strong PPI were found in cluster 2. If we follow crude assumption (a) that all Dey-170 are FLIP (i.e. no true negatives or false positives exist), we still achieve an overall accuracy of 80% (Table S3a). As this assumption is false, this accuracy likely represents a lower limit. Interestingly, though this assumption has a near zero MCC (random guessing) in round 1, subsequent rounds of projection positively improve the correlation to an overall MCC of 0.12. The accuracy and improving MCC suggest that a two-category model, even when mis-assigned is superior to random chance. If we follow crude assumption (b) that weak PPI are FUNC-like and strong PPI are FLIP-like, we obtain results consistent and slightly superior to the training set results with accuracies of 84.7% and an MCC of 0.51 (Table S3b).

As the accuracy and MCC varied somewhat from training set to Test-18 set to Dey-170 set, we evaluated the compositional bias of our training set using random sub-sampling validation (Table S4). Sub-samples of the training set were generated randomly in triplicate for subsets of the training set ranging from 90% down to 20%. Regression analysis at an **α** = 0.10 for 1^st^ through 6^th^ order polynomial fits of number of PPI vs. Accuracy show substantial *Lack of Fit* error and a lack of statistical significance for each. Overall, while this suggests that little compositional bias exists until the number of PPI falls substantially below 80 (50% of the training set), it also suggests that analyzing more PPI will not dramatically improve the overall accuracy.

Taken together these training set and random sub-sampling results suggest our method is robust to protein identity and of general applicability, though likely needing additional refinement in order to boost the accuracy to levels found in other methods [11]–[13].

## Discussion

### ECR analysis can reproducibly distinguish FLIP from FunC interfaces

Through the coupling of biological functional categorization with interface geometries and energetics, the ECR methodology produces very consistent results, both between training and testing sets, as well as between functional sub-categories of PPI. FLIP PPIs can be distinguished from FunC PPIs with 76% accuracy (Table 2). In addition, PPIs of the same functional sub-category generally have similar PC projection values such that they cluster (Figures 4 & S1). An accuracy of 76% compares favorably with other approaches combining several methods [15], [21], [26]. It has slightly lower accuracies (by approximately 10–12%) than PISA, NOXclass, and EPPIC [11]–[13]. While lower in overall accuracy than some of the most accurate methods, it does not appear to have any significant compositional bias. ECR also has a distinct advantage over many methods in that it is based solely on interaction energies and structural features and does not rely on sequence conservation patterns or interactome maps [17]–[19]. However, given the success of approaches like those above that use sequence conservation, particularly sequence entropy, we can expect that future inclusion of features from these other approaches in our analysis would not hinder and might even improve our accuracy. Furthermore, the reproducibility across functional sub-categories, a characteristic not included in the model but rather emergent from the analysis, suggests that this method may also be useful in the annotation of PPIs with unknown function. It is also an improvement on methods that rely solely on hot spot analysis in that through examination of all interface residue interactions it provides an energetic context for the hot spots and their differential presence in FLIP and FunC PPIs.

### Physical Interpretation

From the analysis of CAS ΔΔG energetic and geometric features, several clear patterns emerge. The first of these is that FLIPs appear to have greater overall sensitivity to Ala substitution than FunCs (Figures 3, 4). FLIPs have strong positive correlations with Sum_ΔΔG, #hot, and Avg_ΔΔG in PC1, while FunCs are negatively correlated with these traits (Figure 3b,c and Figure 4). This suggests the FLIPs have more specific interactions that produce large disruptions on Ala substitution than those of FunCs, a finding that agrees with experimental work [1] and is consistent with the characterization of weak and strong interfaces [7].

FLIPs also appear to have larger magnitude feature correlations along PC2 than FunCs, which cluster closer to the PC2 origin. PC2 is dominated by Slope_ΔΔG, intcpt_ΔΔG, and #total (Figure 3b,c), all 3 of which are statistical distinct between FLIP and FunC (P<0.09, P<0.0006, P<0.0001). Taken together, the correlations along PC2 suggest FLIPs have a strong central tendency with hotter centers and more interfacial residues than FunCs. This central tendency of FLIPs is also shown in Figure 1. While superficially, this is in agreement with certain precepts of Bogan and Thorn's “O-ring” hypothesis [8], it helps explain failures of the O-ring hypothesis to explain confounding examples of structures with hydrophilic or mixed hydrophilic and hydrophobic interfaces. A central tendency towards stability could be present in both proteins that follow a hydrophobic O-ring type structure, but could also be present in more hydrophilic interfaces that rely more on solvent and electrostatic interactions.

### Implications for Interface Evolution

The emergence of both a larger specificity and a central organizing tendency from our ECR methodology suggests a model of interface evolution in which nascent, fortuitous interactions in a loose protein-protein association develop residue contacts that improve biological function for the organism. These interactions may have a selective pressure to be maintained or even improved (via mutation) in order to maintain or enhance the specific affinity of the two protein chains (Figure 2c–f). Residues surrounding these contacts may also have pressure to enhance affinity. Over evolutionary time, these selective pressures on the size and specific affinity produce a radially symmetric pattern in the energetics of the interface (Figure 2b,f). The resulting interface should demonstrate “stronger” energies near the “older” regions of the interface. This hypothesis qualitative agrees with the Evolutionary Trace results of Lichtarge and colleagues, who identify radially symmetric “bulls-eye” sequence conservation patterns near functionally important residues [50]. It also helps explain why sequence conservation methods alone without spatial, accessibility, or energetic contributions do not perform well as PPI predictors [15]. As the selective pressure on an interface is on energetic affinity and specificity, not sequence identity, FLIP interfacial residues may actually demonstrate larger sequence variation during the evolutionary “optimization” events. This can occur since improvements in specific affinity could arise if residues in both sides of a PPI were replaced via mutation. Similarly, one would not expect interfaces that are not acted upon by natural selection to have *a priori* central tendency patterns (Figure 2f). They should instead show a more random distribution of important residues (Figure 2c,d).

The ECR concept that evolutionary pressure will produce central tendency patterns with large specificity helps explain some discrepancies in our PCA/K-means cluster data as well. Both Antibody-Antigen and Inhibitor sub-categories cluster near the FunCs and XFunCs in our analysis (Figure 4). While antibody-antigen interactions are decidedly functionally linked, their quaternary structures are generally not evolutionarily driven. Instead, they are produced in a stochastic manner during V(D)J recombination [51]. As somatic cell hypermutation and B-cell selection is an evolutionary-like process [52] and antibody-antigens are minimally oligo-trimers, it is also likely that center of interface of a large oligomer is not near the pairwise center, thus obscuring any central tendency. Similarly, enzyme inhibitors are often produced by infectious organisms to impede a host's native functions. While, the infecting organism may have a selective pressure to improve inhibitor binding, the host organism actually has selective pressures to escape inhibitor binding. For both antibodies and inhibitors, the lack of a *pairwise* central organizing tendency is thus not unlikely and may explain why these two functional sub-categories cluster with the FunCs.

### Implications for Protein Docking

Many protein-docking methods attempt to determine PPI structures by rapidly identifying and scoring regions of complementary shape and electrostatics [40]. Unfortunately, the large false positive rates of most docking methods reduce the usefulness of docking approaches [14], [40], [43]. Presumably, docking calculations are identifying regions of quaternary interaction conformational space that are not accessed by native conformations. As ECR can successfully distinguish FLIP conformations from FunC conformations, we propose ECR's use as a post-filter on the poses resulting from docking calculations. Our preliminary attempts at this look promising. As a proof of concept, we filtered the top 500 scoring poses generated by the docking program Hex [53] with ECR for several Enzymes and Antibody-HL interactions (1tzi_AB, 1bsr_AB, 1bsl_AB, 1biq_AB). In all these, we were able to identify the lowest RMSD pose and in one case, 1bsr_AB, were able to identify a lower RMSD pose than Hex. Though very preliminary, we expect that our ECR method may substantially reduce false positive rates.

### Conclusions

In this work, we have introduced the FLIPdb, a database of protein-protein interfaces categorized by biological function. We have also introduced the Energy Centrality Relationship (ECR) method for analysis of computational alanine scan energetic distributions within protein-protein interfaces. We have successfully identified energetic and geometric features of interfaces that may be used to distinguish between functionally-linked (FLIP) and functionally uncorrelated (FunC) interfaces with a 76–80% accuracy. We have identified that FLIP interfaces have a stronger specificity and central organizing tendency than FunCs. Our ECR model also successfully describes the impact of evolution and natural selection on protein-protein interfaces. Finally, our ECR method may be of use in reducing the false positive rate of docking calculations.

## Methods

### Dataset: FLIPdb

We collected a set of atomic-resolution structures all of which are available in the PDB [32] and then used additional literature and database sources to manually assign protein-protein interfaces to pre-decided categories. The database consists of 94 structures involving 233 individual proteins chains that formed 160 interfaces, which were grouped into two primary categories, functionally-linked (FLIP) or functional uncorrelated (FunC). We initially combined selected subsets of structures from the databases of Janin and Weng [7], [44]–[46]. These datasets characterize proteins by whether they are known to be in protein complexes, have crystal contacts, are weakly or strongly interacting in solution, and how difficult they are to predict. Finally, we supplemented these with structures of general interest in our research. In this work, we chose to expand from prior datasets rather than simply use the datasets outright as these other sets were created to study specific questions but more importantly, did not always clearly delineate biological functional relevance of the PPI. For this work, we limited our selections to only bound complexes in an effort to purposefully limit structural variability and thus bias towards conformations with enhanced specificity. From this initial set, structures with resolutions greater than 3 Å were rejected. We also generally excluded structures with very large cavities or projections whose curvature would produce interface centroids (based on Cα positions) either out in space or far within the interior of one of the binding partners. We further removed any structure with 2 or fewer residues in the interface, partly in an effort to bias towards larger affinities and partly because the use of linear regression to map geometric features requires at least 3 bodies. We rejected structures with disordered residues or heteroatoms other than water or simple ions in the interface in order to bias the analyses towards amino acid interactions.

For each of the resultant interfaces, we performed a limited literature search focused on identifying: (1) whether the proteins were known to oligomerize *in vivo*; (2) whether the proteins were known to oligomerize *in vitro* but under conditions similar to those within living systems; and (3) if mutations, post-translational modification, chemical modification, or small-molecule binding of residues within the interface were known to alter the function of the protein. (4) Additionally, we identified PPIs whose quaternary geometries were generally indicative of biological function, such as cytoskeletal proteins, viral capsid proteins, or immunoglobin interactions between the heavy-chains as well as immunoglobin heavy-chain:light-chain interactions outside the Fv region. We noted, but did not exclusively depend upon, whether PDB/PISA had designated the interface as being present in a Biological Assembly Unit (BioUnit). We categorized interfaces passing all these tests as FLIP. In addition, as a tool to aid our categorization, we noted whether the proteins could be simplistically sub-categorized into: (1) antibody-antigen (AbAg); (2) immunoglobin Heavy Chain/Light Chain (AbHL); (3) Enzyme-Enzyme, both transient and persistent (Enzyme); (4) having a generally persistent structure that provides mechanical stability, such as cytoskeletal or viral proteins (Structural); (5) peptide/protein inhibitors to an enzyme (Inhibitor); (6) proteins whose function is to recognize peptides/proteins (Receptor); or (7) proteins regulated by post-translational modification by another protein (Regulated). We elected to use these 7, admittedly simplistic, operationally-defined sub-categories, rather than use SCOP [54], CATH [55], or GO [56] designations in order to limit the number of sub-categories and thus examine general FLIP characteristics. This is also consistent with categorizing all PPI into only the 2 FLIP/FunC categories. Most interfaces that could not be annotated as FLIP were categorized as FunC, though some interfaces were eliminated from study if a number of conflicting annotations existed.

As the exclusions mentioned previously tended to eliminate FunC structures, we augmented our FunC numbers in two ways. First, we increased the number of proteins with a functionally unrelated PPI in the asymmetric unit by following the inverse of the method of Dey et al.[7]. We supplemented our set with entries from the PiQSi server [39] that were listed as solution-state monomers yet also had an entry of “PROBYES” in the Error field that indicates whether literature is in conflict with the reported quaternary assessment at PDB/PISA. Secondly, we utilized the available crystal symmetries to transform the coordinates of FLIP proteins such that crystal packing contact interfaces were produced. These transformations were created using the SYMEXP module of Pymol [57] and were sub-categorized as XFunCs. While it is generally desirable to have low similarity between dataset members to minimize compositional bias, our use of XFunCs derived from FLIPs actually provides a valuable internal control in that the two should be distinguishable. Failure to distinguish XFunCs from FLIPs in the same protein might suggest that general features of the protein rather than the interface were being biased towards. In order to further increase our FunC structures while maintaining some continuity with the datasets from the literature, we also created XFunCs from a subset of the members of the weakly interacting set of Dey et al. that were listed as only having crystal symmetry. All additional FunCs/XFunCs were also rejected if they failed to pass the same exclusionary limits placed on existing FLIPs and FunCs. In addition, we rejected XFunC structures that literature review suggested might in reality be FLIP. The final database consisted of 94 structures comprised of 219 individual proteins chains that formed 160 interfaces. Of these, 100 were FLIP interfaces and 60 were FunC interfaces. Summary statistics of the FLIPdb are shown in Table 1.

In additional to this training set of interfaces, 18 additional interfaces (Test-18) were analyzed in order to provide a test set for cross-validation. All but two of the proteins in Test-18 had less than 70% sequence identify to proteins in the training set. Identity was determined using BLAST [58] run with default parameters available at servers at the National Center for Biotechnology Information. The remaining 2 proteins (immunoglobin chains), though not identical to immunoglobins in the training set, did have substantial similarity outside of the Fv region. These 18 PPI were subjected to the same physical and literature exclusionary limits as the training set. Summary statistics of the Test-18 are shown in Table 1.

Finally, as the training set had 38 members in common with the set of Dey and colleagues (16 weak and 22 strong in the training set), we created a second cross-validation testing set from 32 additional weak and 138 additional strong interfaces of Dey and colleagues [7]. Dey and colleagues purposefully characterized PPI predicted to have some level of oligomerization in solution, some weakly but most strongly. It is tempting to presume that the majority of these proteins would have some functional importance since they oligomerize in solution. However, without literature curation, one can only assume either (a) that all 170 PPI are FLIP or (b) the strong PPI are more FLIP-like and the weak PPI are more FUNC-like. These assumptions were evaluated in this work. Summary descriptions of these 170 PPI are listed in Table S2.

### Computational Alanine Scanning (CAS)

The CAS method of Kortemme and Baker [29], [30], [59], was used to process all the interfaces in the FLIPdb. In brief, this method evaluates enthalpy and free energy of solvation terms over conformations arising from a rotamer library for both the existing and alanine substituted residues in a PPI (native Gly and Pro excluded). These terms are used to determine a pseudo-free energy change upon substitution (ΔΔG) [30]. Computational Alanine Scanning (CAS) calculations were performed using the Agnito HPC Linux cluster at Texas Woman's University according to scripts and libraries kindly supplied by Dr. Tanja Kortemme (UCSF). These results were spot-checked against CAS calculations made using the ROBETTA server of David Baker's lab [60]. In all cases the results were identical.

### Interfacial Geometry

Interfacial residues were defined using the same interface definition as in the CAS method of Kortemme and Baker [30]. The geometric distribution of residues in each PPI were determined by calculating the displacement (Δr) of the Cα position from the mean of the Cα positions (termed the Center of Interface, CoI) using software written by the authors. A linear regression of the ΔΔG and Δr data to a first-order polynomial (ΔΔG = slope * Δr + intercept) was calculated for each interface using software written by the authors as well as GNUPLOT [61]. The calculations provided 8 features for each interface: the slope (slope_ΔΔG), intercept (intcpt_ΔΔG), coefficient of determination (R2_ΔΔG), net sum of all ΔΔG changes (sum_ΔΔG), mean ΔΔG for all interface residues (avg_ΔΔG), total number of residues in the interface (#total), number of residues with ΔΔG larger than +1 kcal/mol (#hot), and the ratio of “hot” to total (frac_hot). Examples of the distribution of these ΔΔG values for a FLIP (PDBid: 1vfr) and FunC (PDBid: 1c02) are shown in Figure 1.

### Principle Component Analysis (PCA)

Principle component analysis of the variation of CAS energetic and geometric feature data for all PPI was undertaken using JMP [62]. PCA determines a set of linearly-uncoupled eigenvectors from normalized correlations between variables that progressively describe the largest sources of variance in a data set [47]. The eigenvector coefficients for each principal component vector indicate the relative correlation between each feature and the overall variation of all features. In this work, we sought to identify the set of features that would describe more than 80% of the total set variation in the first two principal components (PCs) such that we could use a minimum number of PCs to discriminate between FunC and FLIP data. The results from these PCA analyses are shown in Figures 3 and 4 and Table 2. Due to the lower contribution of the coefficient of determination (R^2^) of the linear regression towards overall feature variation, this term was dropped and only the remaining seven features were used.

### K-means clustering

K-means clustering [48] is a data analysis method that clusters observations into a specific number of clusters by attempting to find the point(s) that have the lowest mean variation from the other input data. When combined with PCA, the combination of features that allows input data to be clustered can be identified. In this work, two clusters were specified and the correlations between cluster and functional category determined (Figure 4a,b and Table 2). Forty-seven (47) FLIP interfaces (mostly enzyme and immunoglobin heavy-chain/light chain interfaces) could easily be identified. A second round of PCA and K-means clustering excluding these 47 FLIP (and 2 FunC PPI falsely identified as FLIP) was subsequently performed (Figure 4c,d and Table 2).

### Accuracy and Matthews Correlation Coefficient

The following measures were used to assess the performance of our clustering analysis:

*Accuracy* (ACC), the propensity to correctly identify FLIP or FunC:
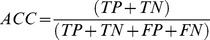
(1)and *Matthews correlation coefficient* (MCC), a measure of how much a set of predictive data agrees with a two-state model:
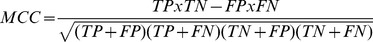
(2)where,

TP  =  the number of interfaces correctly predicted as FLIPs (True Positive)

TN =  the number of interfaces correctly predicted as FunCs (True Negative)

FP  =  the number of interfaces wrongly predicted as FLIPs (False positive)

FN  =  the number of interfaces wrongly predicted as FunCs (False Negative)

These values are shown in Tables 2, S3.

## Supporting Information

Figure S1 **PCA and K-means clustering of Dey-170 set.** Projection of the 7 feature values of the PPI in the Dey-170 set through the principal components developed on the training set. Grey dots show the values of the training set. Green and red ovals represent 1 standard deviation for Euclidean distances around the cluster centroid marked by “**x**”. Values for Dey-170 interfaces are indicated with purple symbols representing “Strong” PPI interactions and blue symbols representing “Weak” PPI interactions. (a) and (b) shows projections through PCA 1 and 2 principal components, respectively. (a) 60% of Strong PPI and 0% Weak PPI group in cluster 1 while 40% of Strong and 100% of Weak group in cluster 2, yielding 100% *precision* and 100% *negative predictive value*. (b) After removal of the 82 PPI in cluster 1, a second projection of the 88 remaining values through PCA 2 produces new clusters with 54 and 34 members, respectively. PCA 2 Cluster 1 is 78% Strong while cluster 2 is 59% Weak. [*Figure generated with JMP* [*46*] *and Microsoft Excel, 2008*].
(TIF)

Table S1 **FLIPdb interface composition.** Structures and interfaces used in the training and testing sets. The FLIPdb database contained 160 pairwise PPI between 219 protein chains that were contained in 94 PDB structural files. The Test-18 set contains 18 pairwise PPI between 19 proteins chains contained in 7 PDB files. Based on literature review, these PPIs were categorized into the FLIP or FunC interface class (100 FLIP, 60 FunC). The PPIs were further sub-categorized into 7 FLIP and 2 FunC sub-categories: 1) antibody-antigen (AbAg); 2) immunoglobin Heavy Chain/Light Chain (AbHL); 3) Enzyme-Enzyme, both transient and persistent (Enzyme); 4) having a generally persistent structure that provides mechanical stability, such as cytoskeletal or viral proteins (Structural); 5) peptide/protein inhibitors to an enzyme (Inhibitor); 6) proteins whose function is to recognize peptides/proteins (Receptor); 7) proteins regulated by post-translational modification by another protein (Regulated); 8) PPIs in an asymmetric crystal unit NOT found to be FLIP (FunC); and 9) PPIs obtained by applying crystal symmetry transforms to FLIP structures (XFunC). The Dey-170 set contains 170 pairwise PPI between 301 proteins chains contained in 139 PDB files. Categories were uncurated and sub-categories of “Strong” and “Weak” were derived from [7]. The number of chains, number of interfaces, and the references used to justify classification for each pairwise interface are listed.
(XLSX)

Table S2 **Summary of protein and protein interface counts in Dey-170.**
(DOCX)

Table S3 **Pseudo-Accuracy of clustering in Dey-170 Test set.**
(DOCX)

Table S4 **Random sub-sample validation of FLIPdb training set.**
(DOCX)
