# Supplementary figures and images for: Protein-Protein Interface Detection Using the Energy Centrality Relationship (ECR) Characteristic of Proteins
Source: PLoS One. 2014 May 15;9(5):e97115. doi: 10.1371/journal.pone.0097115 (PMC4022497; doi:10.1371/journal.pone.0097115)

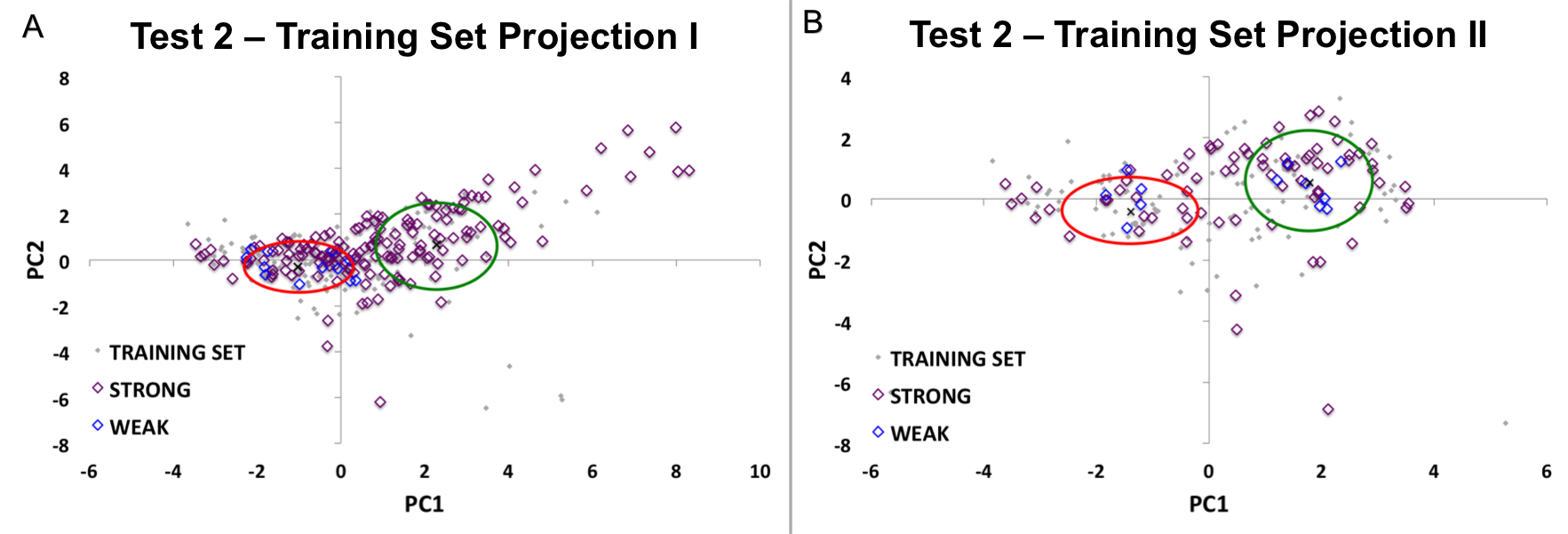

Supplement: Figure S1 — PCA and K-means clustering of Dey-170 set. Projection of the 7 feature values of the PPI in the Dey-170 set through the principal components developed on the training set. Grey dots show the values of the training set. Green and red ovals represent 1 standard deviation for Euclidean distances around the cluster centroid marked by “x”. Values for Dey-170 interfaces are indicated with purple symbols representing “Strong” PPI interactions and blue symbols representing “Weak” PPI interactions. (a) and (b) shows projections through PCA 1 and 2 principal components, respectively. (a) 60% of Strong PPI and 0% Weak PPI group in cluster 1 while 40% of Strong and 100% of Weak group in cluster 2, yielding 100% precision and 100% negative predictive value. (b) After removal of the 82 PPI in cluster 1, a second projection of the 88 remaining values through PCA 2 produces new clusters with 54 and 34 members, respectively. PCA 2 Cluster 1 is 78% Strong while cluster 2 is 59% Weak. [Figure generated with JMP [46] and Microsoft Excel, 2008]. (TIF) [file pone.0097115.s001.tif]
