# Supplementary material for: Protein-Protein Interface Detection Using the Energy Centrality Relationship (ECR) Characteristic of Proteins
Source: PLoS One. 2014 May 15;9(5):e97115. doi: 10.1371/journal.pone.0097115 (PMC4022497; doi:10.1371/journal.pone.0097115)
Supplement: Table S2 — Summary of protein and protein interface counts in Dey-170. (DOCX) [file pone.0097115.s003.docx]

| **Function** | | **Dey-170 Set** | | |
| --- | --- | --- | --- | --- |
| **Category** | **Sub-categories** | **PDB Structures** | **Protein chains*** | **Interfaces** |
| **Unknown** | **Weak** | **17** | **46** | **32** |
|  | **Strong** | **127** | **266** | **138** |
|  | **Total** | **139** | **301** | **170** |

* Proteins chains are common to multiple sub-categories though the interfaces are distinct.

**Table S2. Summary of protein and protein interface counts in Dey-170.**Dey-170 contains 170 interfaces in 139 structures involving 301 individual protein chains. These interfaces have been labeled as Strong or Weak categories based on information available in [7] (see Supplement Table S1). Due to the reuse of some chains, the totals represented in the first two columns do not sum across sub‑categories.
