# Supplementary material for: Protein-Protein Interface Detection Using the Energy Centrality Relationship (ECR) Characteristic of Proteins
Source: PLoS One. 2014 May 15;9(5):e97115. doi: 10.1371/journal.pone.0097115 (PMC4022497; doi:10.1371/journal.pone.0097115)
Supplement: Table S3 — Pseudo-Accuracy of clustering in Dey-170 Test set. (DOCX) [file pone.0097115.s004.docx]

| **Dey-170 Testing Set** | **True Positive (TP)** | **False Positive (FP)** | **False Negative (FN)** | **True Negative (TN)** | **Accuracy** | **MCC** |
| --- | --- | --- | --- | --- | --- | --- |
| a)† **^a^**  Assuming  All:FLIP | **1^st^ clustering projection** | | | | | |
|  | 82 | 0 | 88 | 0 | 48.2% | -0.005 |
|  | **2^nd^ clustering projection** | | | | | |
|  | 54 | 0 | 34 | 0 | 61.4% | 0.05 |
| **Total** | 136 | 0 | 34 | 0 | 80.0% | 0.12 |
| b)‡  Assuming Weak:FUNC Strong:FLIP | **1^st^ clustering projection** | | | | | |
|  | 82 | 0 | 56 | 32 | 67.1% | 0.46 |
|  | **2^nd^ clustering projection** | | | | | |
|  | 42 | 12 | 14 | 20 | 70.4% | 0.37 |
| **Total** | 124 | 12 | 14 | 20 | 84.7% | 0.51 |

† ) TP: FLIP found in Cluster 1 TN: FUNC found in Cluster 2
FP: FUNC found in Cluster 1 FN: FLIP found in Cluster 2

**^a^** ) MCC adjusted to set all zero-values to 1

‡ ) TP: Strong found in Cluster 1 TN: Weak found in Cluster 2
FP: Weak found in Cluster 1 FN: Strong found in Cluster 2

**Table S3: Pseudo-Accuracy of clustering in Dey-170 Test set**
The accuracy and Matthews correlation coefficient of the results of the cluster projections for the PPI in the Dey-170 test set shown in Figure S1. Values likely represent a lower limit, as all PPI in this set were purposely not rigorously curated and were operationally presumed (a) to be FLIP or (b) for Weak to be FUNC and Strong interactions to be FLIP. (a) The overall accuracy is 80%. As expected from eliminating FP and TN, the MCC of the 1^st^ clustering is near zero (random guessing). Interestingly, subsequent projection rounds and the overall MCC shift positively to 0.12. The accuracy greater than 75% and MCC > 0 suggest that a two-category model is still more generally appropriate than random guessing even with crude assumptions. (b) Presuming Weak interactions match FUNC and Strong interactions match FLIP yields a larger accuracy of 84.7% and a MCC of 0.51.
