# Supplementary material for: Protein-Protein Interface Detection Using the Energy Centrality Relationship (ECR) Characteristic of Proteins
Source: PLoS One. 2014 May 15;9(5):e97115. doi: 10.1371/journal.pone.0097115 (PMC4022497; doi:10.1371/journal.pone.0097115)
Supplement: Table S4 — Random sub-sample validation of FLIPdb training set. (DOCX) [file pone.0097115.s005.docx]

| **% FLIPdb (# of Interfaces)** | **Trial** | **TP†** | **FP†** | **FN†** | **TN†** | **Accuracy** | **MCC** |
| --- | --- | --- | --- | --- | --- | --- | --- |
| 100 (160) | **1** | **80** | **18** | **20** | **42** | **76%** | **0.50** |
| 90 (144) | **1** | **68** | **15** | **21** | **40** | **75%** | **0.48** |
|  | **2** | **70** | **16** | **19** | **39** | **76%** | **0.49** |
|  | **3** | **74** | **18** | **17** | **36** | **76%** | **0.48** |
| 80 (128) | **1** | **67** | **19** | **11** | **31** | **77%** | **0.50** |
|  | **2** | **64** | **15** | **16** | **33** | **76%** | **0.49** |
|  | **3** | **64** | **14** | **19** | **30** | **74%** | **0.44** |
| 70 (112) | **1** | **32** | **0** | **35** | **45** | **69%** | **0.52** |
|  | **2** | **56** | **16** | **12** | **28** | **75%** | **0.47** |
|  | **3** | **40** | **3** | **29** | **40** | **71%** | **0.51** |
| 60 (96) | **1** | **39** | **4** | **22** | **31** | **73%** | **0.51** |
|  | **2** | **47** | **15** | **12** | **22** | **72%** | **0.40** |
|  | **3** | **53** | **14** | **12** | **17** | **73%** | **0.37** |
| 50 (80) | **1** | **38** | **2** | **14** | **26** | **80%** | **0.63** |
|  | **2** | **34** | **4** | **17** | **23** | **73%** | **0.49** |
|  | **3** | **42** | **10** | **12** | **16** | **73%** | **0.39** |
| 40 (64) | **1** | **15** | **3** | **20** | **26** | **64%** | **0.36** |
|  | **2** | **30** | **12** | **8** | **14** | **69%** | **0.34** |
|  | **3** | **25** | **4** | **14** | **21** | **72%** | **0.47** |
| 30 (48) | **1** | **20** | **1** | **15** | **12** | **67%** | **0.44** |
|  | **2** | **18** | **1** | **11** | **18** | **75%** | **0.57** |
|  | **3** | **19** | **4** | **7** | **16** | **76%** | **0.53** |
| 20 (32) | **1** | **15** | **2** | **4** | **11** | **81%** | **0.63** |
|  | **2** | **12** | **5** | **7** | **8** | **63%** | **0.24** |
|  | **3** | **10** | **0** | **15** | **7** | **53%** | **0.36** |

† ) TP: FLIP found in Cluster 1 TN: FUNC found in Cluster 2
FP: FUNC found in Cluster 1 FN: FLIP found in Cluster

**Table S4: Random sub-sample validation of FLIPdb training set**
The distribution of overall accuracies and MCCs of repeated retraining when sub‑samples of the training set were generated randomly in triplicate for subsets ranging from 90% to 20% of the original. The general accuracy is 70-80% until more than 50% of the training set is removed. MCCs stably range between approximately 0.20-0.60. This stability suggests little compositional bias in the FLIPdb training set.
